# Serum GDF15 level as predictive biomarker of clinical outcome in patients with unresectable hepatocellular carcinoma treated with hepatic arterial infusion chemotherapy

**DOI:** 10.3389/fimmu.2025.1619387

**Published:** 2025-07-03

**Authors:** Rui Xing, Junyu Gan, Jie Mei, Zhixiong Li, Jing Xu

**Affiliations:** ^1^ State Key Laboratory of Oncology in South China, Guangdong Provincial Clinical Research Center for Cancer, Sun Yat-sen University Cancer Center, Guangzhou, China; ^2^ Department of Liver Surgery, Sun Yat-sen University Cancer Center, Guangzhou, China

**Keywords:** growth differentiation factor 15, hepatocellular carcinoma, hepatic arterial infusion chemotherapy, serum biomarker, overall survival, progression-free survival

## Abstract

**Background:**

Hepatic arterial infusion chemotherapy (HAIC) using the FOLFOX regimen has been explored for unresectable hepatocellular carcinoma (HCC) patients, yet predictive biomarkers are lacking. This study aimed to evaluate the potential of serum growth differentiation factor 15 (GDF15) as a biomarker for predicting therapeutic response and survival outcomes in HCC patients undergoing FOLFOX-HAIC.

**Methods:**

Pretreatment serum samples were collected from patients with unresectable HCC who received FOLFOX-HAIC between October 2016 and January 2019. GDF15 levels were measured using enzyme-linked immunosorbent assay (ELISA). Associations between serum GDF15 levels and treatment response, overall survival (OS), progression-free survival (PFS), and clinical characteristics were analyzed.

**Results:**

A total of 150 patients were included in the study. The mean GDF15 level was 7.16 ng/mL (mean ± SEM: 7.16 ± 0.72; range: 0.39-53.55 ng/mL). High serum GDF15 levels were significantly associated with poorer treatment response, shorter OS (median: 21.1 vs 40.33 months, *p* = 0.0081) and PFS (median: 13.93 vs 20.47 months, *p* = 0.0125). Multivariate Cox proportional hazards analysis identified serum GDF15 as an independent predictor of PFS (HR, 1.521; 95% CI, 1.014-2.283; *p* = 0.043). Additionally, elevated GDF15 was positively correlated with larger tumor size (*p* < 0.0001), presence of microvascular invasion (*p* = 0.026) and abnormal AST levels (*p* = 0.001).

**Conclusion:**

Serum GDF15 represents a potential prognostic biomarker in patients with unresectable HCC undergoing FOLFOX-HAIC treatment and may help guide treatment stratification.

## Introduction

Hepatocellular carcinoma (HCC) is one of the most prevalent malignancies and the fourth leading cause of cancer-related mortality worldwide (1–3). Most HCC patients are diagnosed at intermediate or advanced stages, when curative treatments such as tumor resection or liver transplantation are no longer viable (4). For unresectable HCC, systemic therapy remains the primary treatment approach (5, 6). The combination of immune checkpoint inhibitors (ICIs) with tyrosine kinase inhibitors (TKIs) or other agents has demonstrated overall survival (OS) benefits. However, the response rate of ICI-based therapies remains relatively low (20%-30%), underscoring the need for novel strategies to improve treatment efficacy (7).

Recent clinical trials have shown that hepatic arterial infusion chemotherapy (HAIC), which delivers high local concentrations of oxaliplatin, leucovorin, and fluorouracil (FOLFOX) without embolization, can improve clinical outcomes in patients with unresectable HCC (8–10). Several retrospective studies and clinical trials suggest that HAIC offer superior efficacy compared to transarterial chemoembolization and sorafenib (11–14). Moreover, the therapeutic potential of HAIC may be further enhanced when combined with ICIs and/or TKIs, supporting FOLFOX-HAIC as a promising treatment strategy for unresectable HCC (15–17). However, the response rate of HAIC ranges from 25% to 46%, suggesting that its efficacy still needs to be improved. Moreover, the considerable variability in treatment response underscores the need for predictive biomarkers to guide personalized and cost-effective treatment decisions.

Growth differentiation factor 15 (GDF15), a cytokine belonging to the TGF-β superfamily, plays a crucial role in cell activation and stress response (18). Elevated serum GDF15 levels have been observed in various conditions, including diabetes, metabolic syndrome, and anorexia, and liver diseases such as cirrhosis and non-alcoholic fatty liver disease (19). In the context of cancer, GDF15 has been shown to promote tumor cell proliferation, enhance resistance to apoptosis, and contribute to an immunosuppressive tumor microenvironment, thereby facilitating tumor progression (20, 21). Aberrant expression of GDF15 has been reported in multiple malignances including liver cancer, and is associated with patient prognosis (22, 23). However, its role in predicting treatment response and clinical outcomes of HCC patients receiving HAIC remains to be elucidated.

In this study, we analyzed serum GDF15 levels in HCC patients prior to HAIC and found that higher GDF15 levels were associated with poorer treatment response, shorter OS and reduced progression-free survival (PFS), highlighting its potential as a prognostic biomarker for unresectable HCC undergoing HAIC.

## Materials and methods

### Patients

From October 2016 to January 2019, the clinical records of 150 patients with primary HCC who underwent HAIC at Sun Yat-sen University Cancer Center were retrospectively collected. Tumor staging was assessed according to the Barcelona Clinic Liver Cancer (BCLC) staging system. Unresectable HCC was defined as BCLC stage B or C disease, or BCLC A cases deemed unsuitable for surgery due to tumor location, liver function, or comorbidities (24, 25). The inclusion criteria were as follows: no prior treatment before HAIC, a clinically or pathologically diagnosis of HCC, available follow-up data, and Child–Pugh class A or B liver function. Baseline clinical characteristics prior to the first HAIC cycle was recorded. This study complies with the principles of the Declaration of Helsinki and was approved by the Institutional Review Board of Sun Yat-Sen University Cancer Center.

### Treatment

The detail procedure, dosage modification criteria, and management of infusion-related reactions for FOLFOX-HAIC treatment were performed as described in previous studies (10–12). Briefly, oxaliplatin (130 mg/m^2^) was administered over 2 hours on day 1 (hour 0 to 2), followed by leucovorin (400 mg/m^2^) over 1 hour (hour 2 to 3), then a bolus of fluorouracil (400 mg/m^2^) at hour 3, and a continuous infusion of fluorouracil (2400 mg/m^2^) over the subsequent 24 hours. Patients generally received 4 cycles of HAIC administered at 4–5 weeks intervals.

### Follow-up

Patients were monitored following HAIC treatment, with follow-up assessments conducted every 6 months until disease progression or death. Regular evaluations included dynamic computed tomography scans and magnetic resonance imaging, routine blood tests, liver function tests, and tumor marker analyses. OS was defined as the time from initial treatment to death or last follow-up. PFS was defined as the time from treatment initiation to either disease progression or death from any cause. Treatment response was evaluated according to RECIST version 1.1. The median follow-up duration was 24.5 months, with the last follow-up conducted in December 2024.

### Serum GDF15 level examination

Serum samples were collected from patients before their first HAIC treatment and stored at −80°C until analysis. GDF15 concentrations were measured using enzyme-linked immunosorbent assay (ELISA) kits (Sino Biological), according to the manufacturer’s instructions.

### Statistical analysis

Data analysis and visualization were performed using IBM SPSS Statistics (v25), GraphPad Prism (v8.0.2) and R (v4.2.2). Group comparisons were analyzed using Student’s *t*-test for continuous variables. Categorical variables were analyzed using the Chi-square test or Fisher’s exact test, as appropriate. Survival curves were generated using the Kaplan–Meier method, and statistical comparisons were conducted using the log rank test. Hazard ratio (HR) and 95% confidence interval (CI) were estimated using univariate and multivariate Cox proportional hazards models. A two-sided *p*-value < 0.05 was considered statistically significant.

## Results

### Clinical characteristics

**Figure 1:**
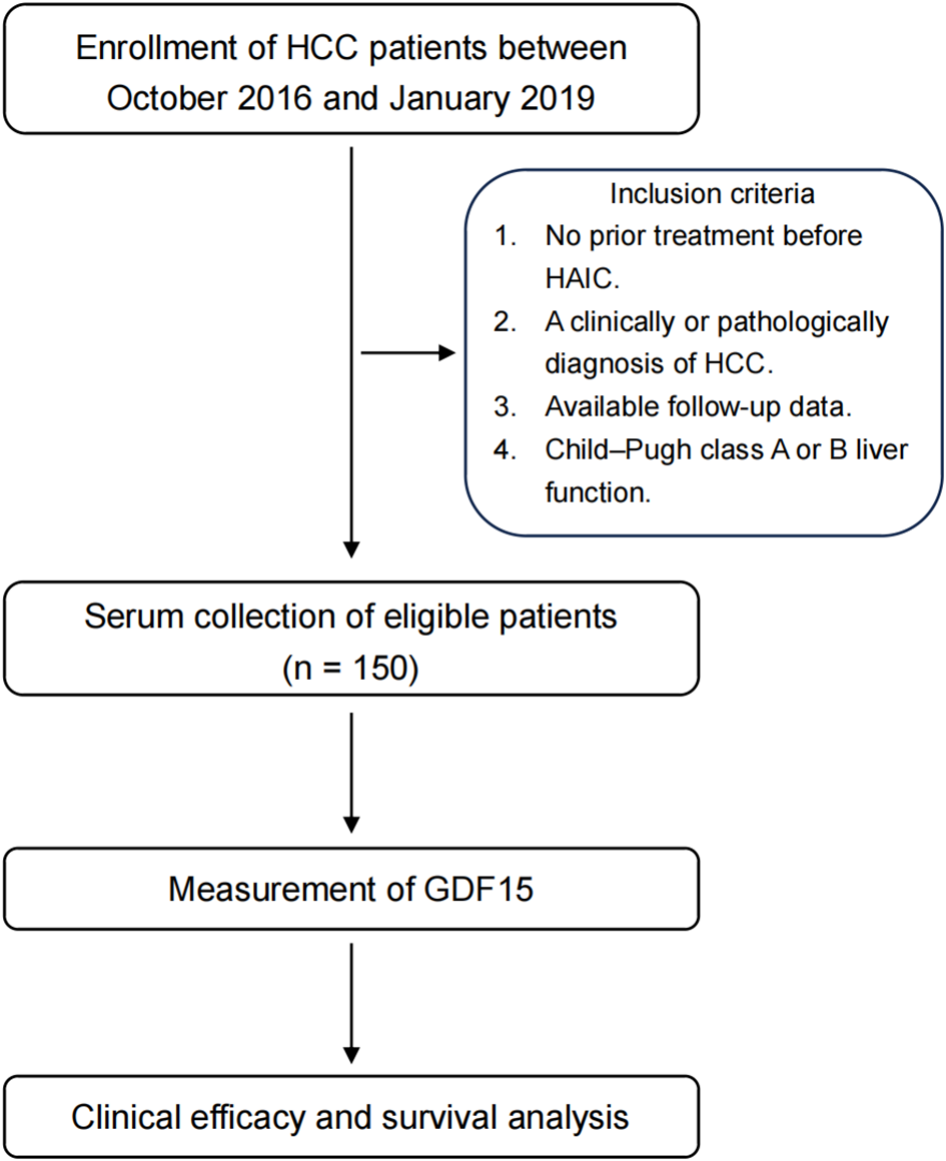
Study design. Brief graphic study design of this study. HCC, hepatocellular carcinoma; HAIC, hepatic arterial infusion chemotherapy; GDF15, growth differentiation factor 15.

Between October 2016 and January 2019, a total of 150 patients who received HAIC treatment for primary HCC were enrolled in this study (**Figure 1**). The baseline clinical characteristics of all patients are summarized in **Table 1**. The median age was 53 years, and 84.67% of patients were male. Hepatitis B virus (HBV) infection was present in 79.33% of cases. Elevated alpha-fetoprotein (AFP) levels (> 400 ng/ml) were observed in 48% of patients, while elevated alanine aminotransferase (ALT > 40 U/L) and aspartate aminotransferase (AST > 40 U/L) levels were found in 55.33% and 74.67% of patients, respectively. Moreover, 48% of patients presented with multiple lesions, 49.19% had microvascular invasion, and 27.33% had extrahepatic metastasis. More than half of the patients (52.67%) were classified as BCLC stage C (**Table 1**). Additionally, the median level of neutrophil-to-lymphocyte ratio (NLR) was 2.71, and 59.33% of patients had elevated C-reactive protein (CRP > 5 mg/L). The average level of serum GDF15 was 7.16 ng/mL (mean ± SEM, 7.16 ± 0.72), with a range of 0.39 to 53.55 ng/mL.

**Table 1:** Baseline characteristics of patients.

Characteristics		No. of patients (%)
Age, years, median (range)		53 (13 - 80)
Sex	male	127 (84.67%)
	female	23 (15.33%)
Tumor size	≥ 10cm	64 (42.67%)
	< 10cm	86 (57.33%)
Tumor number	multiple	72 (48.00%)
	single	78 (52.00%)
Microvascular invasion*	yes	61 (49.19%)
	no	63 (50.81%)
Extrahepatic metastasis	yes	41 (27.33%)
	no	109 (72.67%)
AFP (ng/ml)	> 400	72 (48.00%)
	≤ 400	78 (52.00%)
BCLC stage	A	43 (28.67%)
	B	28 (18.66%)
	C	79 (52.67%)
HBsAg	yes	119 (79.33%)
	no	31 (20.67%)
Cirrhosis	yes	43 (28.67%)
	no	107 (71.33%)
ALT (U/L)	> 40	83 (55.33%)
	≤ 40	67 (44.67%)
AST (U/L)	> 40	112(74.67%)
	≤ 40	38 (25.33%)
NLR, median (95%CI)		2.71 (2.98-4.67)
CRP (mg/L)	> 5	89 (59.33%)
	≤ 5	61 (40.67%)
Serum GDF15 (ng/mL), mean ± SEM (range)		7.16 ± 0.72 (0.39 - 53.55)

AFP, alpha-fetoprotein; ALT, alanine aminotransferase; AST, aspartate aminotransferase; BCLC, Barcelona Clinic Liver Cancer; NLR, neutrophil-to-lymphocyte ratio; CRP, C-reactive protein; GDF15, growth differentiation factor 15.

*Data is missing for some patients.

### Correlation between serum GDF15 level and therapeutic response

**Figure 2:**
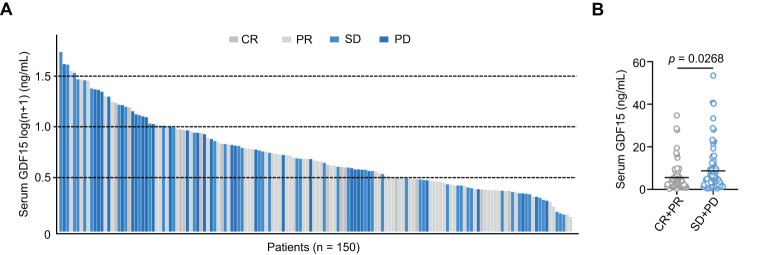
Serum GDF15 high levels are associated with poor clinical efficacy. **(A)** Bar plot showing serum GDF15 levels in all enrolled patients after HAIC treatment (n = 150). **(B)** Bar plot comparing serum GDF15 levels between responders (n = 73) and non-responders (n = 77). *P* values were calculated using the student’s t test. GDF15, growth differentiation factor 15.

HCC patients with higher serum GDF15 tended to have poorer clinical responses (stable disease, SD; and progressive disease, PD), whereas patients who achieved a complete response (CR) or partial response (PR) generally had lower serum GDF15 levels (**Figure 2A**). Consistently, non-responders (SD and PD, n = 77) had significantly higher serum GDF15 levels compared to responders (PR and CR, n = 73; **Figure 2B**). These results suggest that higher serum GDF15 levels are associated reduced clinical efficacy in patients undergoing HAIC.

### Correlation between serum GDF15 level and patient prognosis

**Figure 3:**
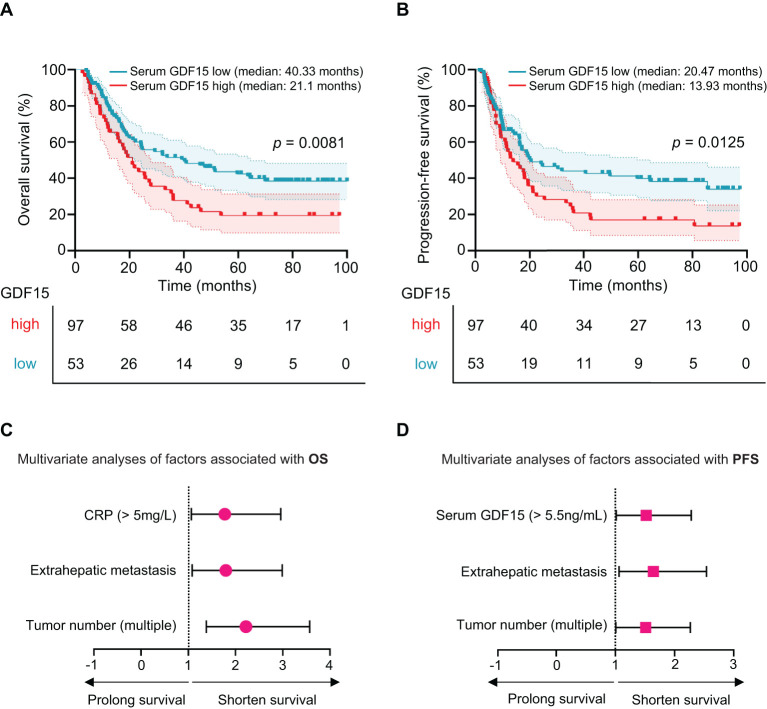
Serum GDF15 high levels are associated poor survival in HCC patients. **(A, B)** Kaplan-Meier plot showing overall survival (OS, **A**) and progression-free survival (PFS, **B**) for HCC patients with high (n = 53) and low (n = 97) serum GDF15 levels after HAIC treatment. *P* values were calculated using the log-rank test. **(C, D)** Multivariate Cox proportional hazards analysis of statistically significant clinical characteristics in HAIC-treated HCC patient for OS **(C)** and PFS **(D)**. Hazard ratios with 95% confidence intervals are shown. Red indicates high-risk factors. GDF15, growth differentiation factor 15.

To assess the prognostic value of serum GDF15 in HCC patients receiving HAIC, patients were divided into high (range: 5.76-53.55 ng/mL) and low (range: 0.39-5.47 ng/mL) GDF15 groups based on the optimal cut-off value determined by the minimum *P*-value method for overall survival (OS). Patients with high GDF15 levels had a significantly shorter median OS of 21.1 months, compared to 40.33 months in the low GDF15 group (*p* = 0.0081; **Figure 3A**). Similarly, the median progression free survival (PFS) was shorter in the high GDF15 group (13.93 months) than in the low GDF15 group (20.47 months, *p* = 0.0125; **Figure 3B**). These results indicate that elevated serum GDF15 levels are associated with poorer survival outcomes in HCC patients treated with HAIC.

### Serum GDF15 was an independent prognostic factor for HCC

Univariate analysis showed that tumor number (*p* = 0.0001), microvascular invasion (*p* = 0.014), extrahepatic metastasis (*p* = 0.002), AST (*p* = 0.034*)*, CRP *(p =* 0.003), BCLC stage C (*p* = 0.038), and serum GDF15 (*p* = 0.009) were significantly associated with OS (**Table 2**). For PFS, the correlative factors were tumor number (*p* = 0.018), extrahepatic metastasis (*p* = 0.005), and serum GDF15 (*p* = 0.013; **Table 3**).

**Table 2:** Univariate and multivariate analyses of factors associated with OS.

Characteristic	Univariate			Multivariate		
	HR	95% CI	*p*	HR	95% CI	*p*
Sex (male)	1.312	0.745-2.313	0.347			
Tumor size (≥ 10 cm)	1.356	0.912-2.016	0.133			
Tumor number (multiple)	2.151	1.432-3.232	0.0001	2.221	1.382-3.571	0.001
Microvascular invasion (yes)*	1.785	1.122-2.841	0.014			
Extrahepatic metastasis (yes)	1.928	1.269-2.928	0.002	1.797	1.081-2.987	0.024
AFP (> 400 ng/ml)	0.745	0.630-1.392	0.936			
BCLC stage (C)	1.534	1.023-2.299	0.038			
HBsAg (yes)	1.067	0.646-1.762	0.799			
Cirrhosis (yes)	1.149	0.744-1.773	0.530			
ALT (> 40 U/L)	1.304	0.870-1.954	0.198			
AST (> 40 U/L)	1.723	1.043-2.844	0.034			
NLR (> 2.7)	1.486	0.998-2.214	0.051			
CRP (> 5 mg/L)	1.886	1.238-2.872	0.003	1.775	1.065-2.957	0.028
Serum GDF15 (> 5.5 ng/mL)	1.714	1.145-2.566	0.009			

Variables associated with overall survival (OS) by univariate analysis were adopted as covariates in multivariate analysis and entered into the equation by the forward selection based on likelihood ratio test. A Bold indicate significance of *p* value (*p* < 0.05).

AFP, alpha-fetoprotein; ALT, alanine aminotransferase; AST, aspartate aminotransferase; BCLC, Barcelona Clinic Liver Cancer; NLR, neutrophil-to-lymphocyte ratio; CRP, C-reactive protein; GDF15, growth differentiation factor 15; HR, hazard ratio; CI, confidence interval. *Data is missing for some patients.

**Table 3:** Univariate and multivariate analyses of factors associated with PFS.

Characteristic	Univariate			Multivariate		
	HR	95% CI	*p*	HR	95% CI	*p*
Sex (male)	1.172	0.653-2.102	0.595			
Tumor size (≥ 10 cm)	1.415	0.950-2.109	0.088			
Tumor number (multiple)	1.627	1.087-2.434	0.018	1.512	1.008-2.268	0.046
Microvascular invasion (yes)*	1.432	0.903-2.272	0.127			
Extrahepatic metastasis (yes)	1.858	1.211-2.852	0.005	1.644	1.064-2.540	0.025
AFP (> 400 ng/ml)	0.777	0.522-1.159	0.216			
BCLC stage (C)	1.479	0.987-2.217	0.058			
HBsAg (yes)	1.082	0.647-1.807	0.765			
Cirrhosis (yes)	1.076	0.690-1.678	0.747			
ALT (> 40 U/L)	1.206	0.802-1.814	0.367			
AST (> 40 U/L)	1.318	0.812-2.137	0.264			
NLR (> 2.7)	1.467	0.983-2.190	0.061			
CRP (> 5 mg/L)	1.381	0.916-2.082	0.124			
Serum GDF15 (> 5.5 ng/mL)	1.663	1.114-2.483	0.013	1.521	1.014-2.283	0.043

Variables associated with progression-free survival (PFS) by univariate analysis were adopted as covariates in multivariate analysis and entered into the equation by the forward selection based on likelihood ratio test. A Bold indicate significance of *p* value (*p* < 0.05).

AFP, alpha-fetoprotein; ALT, alanine aminotransferase; AST, aspartate aminotransferase; BCLC, Barcelona Clinic Liver Cancer; NLR, neutrophil-to-lymphocyte ratio; CRP, C-reactive protein; GDF15, growth differentiation factor 15; HR, hazard ratio; CI, confidence interval. * Data is missing for some patients.

Multivariate analysis was further employed, and the results showed that tumor number (HR, 2.221; 95% CI, 1.382-3.571; *p* = 0.001), extrahepatic metastasis (HR, 1.797; 95% CI, 1.081-2.987; *p* = 0.024), and CRP (HR, 1.775; 95% CI, 1.065-2.957; *p* = 0.028) were independently associated with OS (**Table 2**, **Figure 3C**). For PFS, tumor number (HR, 1.512; 95% CI, 1.008-2.268; *p* = 0.046), extrahepatic metastasis (HR, 1.644; 95% CI, 1.064-2.540; *p* = 0.025), and serum GDF15 (HR, 1.521; 95% CI, 1.014-2.283; *p* = 0.043) were independent predictors (**Table 3**, **Figure 3D**). Together, these results suggest that serum GDF15 serves as a prognostic biomarker for disease progression in unresectable HCC following HAIC treatment.

### Correlations between serum GDF15 levels and clinical characteristics

Patients with high serum GDF15 levels had a significantly higher proportion of larger tumors (≥ 10cm, *p* < 0.0001), microvascular invasion (*p* = 0.026), and abnormal AST levels (*p* = 0.001), compared to those with low serum GDF15 levels (**Table 4**). Besides, serum GDF15 levels were negatively associated with cirrhosis incidence (*p* = 0.019). No significant differences were observed between the two groups regarding sex, tumor number, extrahepatic metastasis, AFP levels, BCLC stage, or HBsAg status. In addition, serum GDF15 levels showed no significant correlation with ALT, NLR, or CRP levels. These data suggest that high serum GDF15 levels are associated with increased tumor size and microvascular invasion, potentially contributing to HCC progression.

**Table 4:** Correlation between serum GDF15 level and clinical characteristics.

Variable	Serum GDF15 low	Serum GDF15 high	*p* value
Sex (male)	84/97 (86.60%)	43/53 (81.13%)	0.374
Tumor size (≥ 10cm)	31/97 (31.96%)	33/53 (62.26%)	< 0.0001
Tumor number (multiple)	44/97 (45.36%)	28/53 (52.83%)	0.381
Microvascular invasion (yes)*	35/83 (42.17%)	26/41 (63.41%)	0.026
Extrahepatic metastasis (yes)	23/97 (23.71%)	18/53 (33.96%)	0.178
AFP (> 400 ng/ml)	50/97 (51.55%)	28/53 (52.83%)	0.880
BCLC stage (C)	46/97 (47.42%)	33/53 (62.26%)	0.253
HBsAg (yes)	79/97 (81.44%)	40/53 (75.47%)	0.320
Cirrhosis (yes)	34/97 (35.05%)	9/53 (16.98%)	0.019
ALT (> 40 U/L)	50/97 (51.55%)	33/53 (62.26%)	0.207
AST (> 40 U/L)	64/97 (65.98%)	48/53 (90.57%)	0.001
NLR (> 2.71)	43/97 (44.33%)	32/53 (60.38%)	0.087
CRP (> 5 mg/mL)	52/97 (53.61%)	37/53 (69.81%)	0.053

The correlations between serum GDF15 level and other clinical characteristics were detected by the Chi-square test.

AFP, alpha-fetoprotein; ALT, alanine aminotransferase; AST, aspartate aminotransferase; BCLC, Barcelona Clinic Liver Cancer; NLR, neutrophil-to-lymphocyte ratio; CRP, C-reactive protein; GDF15, growth differentiation factor 15; HR, hazard ratio; CI, confidence interval. *Data is missing for some patients.

## Discussion

In this study, we investigated the prognostic significance of serum GDF15 levels in unresectable HCC undergoing HAIC. Our results demonstrated that patients with higher serum GDF15 levels before HAIC treatment had poorer therapeutic responses and shorter OS and PFS compared to those with lower levels. Multivariate Cox regression analysis further revealed that serum GDF15 was an independent factor of PFS. These findings suggest that GDF15 may serve as a potential biomarker for predicting HAIC outcomes and guiding treatment decisions in unresectable HCC patients.

FOLFOX-HAIC is an emerging treatment approach for unresectable and advanced HCC patients, with response rates ranging from 25% to 46% (26). Several studies have developed prognostic models to improve patient stratification for HAIC. For instance, models incorporating clinical variables or machine-learning-based model have shown improved survival prediction compared to traditional TNM staging (27–30). These studies highlight the need for predictive biomarkers to guide treatment selection. Circulating biomarkers are essential for clinical diagnosis, disease monitoring, therapeutic efficacy prediction, and prognosis assessment in patients (31, 32). Common biomarkers such as AFP, CRP, and NLR are widely used in clinical practice, and have also been explored in HAIC-treated patients (33, 34). In this study, a high serum CRP level was independently associated with OS, suggesting its potential as a prognostic marker for HCC patients undergoing HAIC.

GDF15 is a dimeric protein of 224 amino acids, stabilized by a conserved inter-chain disulfide bond, with a molecular weight of approximately 25 KDa (35). Its stable presence in circulation enhances its reliability as a serum biomarker. In healthy individuals, circulating GDF15 levels range from 200 to 1200 pg/mL, but can increase by 10- to 100-fold under conditions such as aging, pregnancy, injury, inflammation, and neoplasia (36). Notably, evaluated serum GDF15 levels have been reported in patients with cirrhosis (mean ± SEM: 6.51 ± 1.47 ng/mL) and HCC (mean ± SEM: 6.66 ± 0.67 ng/mL) (37). In this study, the mean of serum GDF15 in unresectable HCC patients was 7.16 ng/mL (range: 0.39-53.55 ng/mL), further supporting its potential as a biomarker. Recently research has shown that Visugromab, a GDF15 neutralizing antibody, can improve efficacy of anti-PD-1-based cancer immunotherapy in non-squamous non-small cell lung cancer, urothelial cancer, and potentially HCC, highlighting its clinical relevance (38). Our results indicate that elevated pre-treatment serum GDF15 levels are associated with reduced clinical efficacy and shorter PFS in patients undergoing HAIC. This suggests that GDF15-neutralizing strategies may improve treatment outcomes. A potential design for future clinical trials could include an initial safety run-in phase using a GDF15-neutralizing antibody alone, followed by combination therapy with HAIC. Moreover, since serum GDF15 levels may be influenced by HAIC treatment, monitoring these levels dynamically could serve as a potential biomarker to evaluate therapeutic response.

As a member of the TGF-β superfamily, GDF15 holds potential prognostic value and has been shown to improve the clinical efficacy of immunotherapy, prompting us to investigate its clinical significance in patients treated with HAIC (38, 39). GDF15 is expressed by various cell types in the liver and HCC tissues, including hepatic stellate cells, macrophages, tumor cells, and hepatocytes. For example, in mouse models of non-alcoholic steatohepatitis, downregulated ARRB1 in hepatocytes impairs the transport of GDF15 precursor to the Golgi apparatus for cleavage and maturation, thereby promoting intracellular lipid accumulation (40). In liver cancer models, hepatic stellate cells secrete GDF15 to stimulate hepatoma cell proliferation, while HCC-derived GDF15 facilitates the generation of inducible Treg cells, contributing to an immunosuppressive tumor microenvironment (41, 42). The tumor microenvironment comprises a complex network of cellular components, which collectively influence tumor progression (43–45). Our study explored the correlation between GDF15 and systemic inflammatory markers such as CRP and NLR; however, no significant association was observed.

There are serval limitations in our study. First, the lack of matched pre- and post-treatment samples restricted our ability to explore the temporal dynamics of GDF15 levels during HAIC. Second, the *in situ* expression and mechanistic role of GDF15 during HAIC treatment remain unclear, highlighting the need for further investigation. Third, a risk stratification model may help stratify HCC patients into clinically relevant risk categories to better guide treatment decisions.

In summary, serum GDF15 was identified as a prognostic biomarker in unresectable HCC patients undergoing HAIC treatment. Targeting GDF15 may represent a potential therapeutic strategy to enhance HAIC efficacy and improve patient survival.

## Data Availability

The original contributions presented in the study are included in the article/Supplementary Material. Further inquiries can be directed to the corresponding authors.
